# Intraoperative Detection and Management of Euglycemic Ketonemia During Emergency Surgery Using Point-of-Care Blood Beta-Hydroxybutyrate Testing in a Patient Receiving Sodium-Glucose Cotransporter 2 (SGLT2) Inhibitors: A Case Report

**DOI:** 10.7759/cureus.100409

**Published:** 2025-12-30

**Authors:** Hiroshi Takanami, Kozue Kubo, Sachiyo Sakamoto, Munehiro Masuzawa, Takahiko Kamibayashi

**Affiliations:** 1 Anesthesiology, Kansai Medical University Medical Center, Osaka, JPN; 2 Anesthesiology and Critical Care, Kansai Medical University Medical Center, Osaka, JPN

**Keywords:** blood beta-hydroxybutyrate, diabetic ketoacidosis, empagliflozin, inadequate preoperative duration of discontinuation, intraoperative management, normoglycemic ketosis, sodium‒glucose cotransporter 2 inhibitors

## Abstract

Patients who receive sodium-glucose cotransporter 2 inhibitors (SGLT2is) are at risk of developing euglycemic ketoacidosis (EDKA), particularly during the perioperative period, if drug withdrawal is insufficient. Although current guidelines recommend withholding SGLT2is prior to surgery, adequate withdrawal periods cannot always be ensured in emergency situations. However, to date, no standardized intraoperative management strategies have been established to address this risk in such situations. To address this gap, we developed a prototype intraoperative management regimen at our institution specifically for patients undergoing emergency surgery without sufficient SGLT2i withdrawal. This regimen was established on the basis of previously reported strategies for managing EDKA and included point-of-care blood beta-hydroxybutyrate (BHB) testing as a key intraoperative monitoring tool. In this case report, we describe the successful application of this regimen in an emergency surgical setting, resulting in stable intraoperative metabolic control without the development of ketoacidosis.

## Introduction

Sodium-glucose cotransporter 2 inhibitors (SGLT2is) are drugs used to manage type 2 diabetes and suppress glucose reabsorption from primary urine via SGLT2, which is specifically expressed in renal tubules, thereby lowering blood glucose concentrations [1]. In addition, these drugs have also been reported to exert cardioprotective and renoprotective effects [2] and are used to treat chronic heart failure and chronic kidney disease. In recent years, it has been reported that some patients who are treated with SGLT2is have developed diabetic ketoacidosis (DKA) or euglycemic ketoacidosis (EDKA) [3], and a reduction in insulin administration, poor dietary intake, and fasting during SGLT2i administration are considered exacerbating factors [4].

At our institution, SGLT2is are routinely discontinued three days prior to surgery to reduce the risk of intraoperative DKA. However, in certain emergency situations, a sufficient preoperative SGLT2i withdrawal period cannot always be ensured. In such cases of inadequate withdrawal, the administration of glucose and insulin is considered necessary to prevent the development of DKA. However, an appropriate intraoperative dosing regimen, particularly under general anesthesia, has not yet been established. To address this, we developed an intraoperative management regimen in which glucose and insulin were administered based on serial measurements of the beta-hydroxybutyrate (BHB) levels.

In this report, we describe the intraoperative management of a patient who underwent emergency surgery and presented with preoperative ketonemia due to insufficient SGLT2i withdrawal. By applying our regimen, we successfully prevented progression from ketonemia to ketoacidosis during anesthesia and surgery.

## Case presentation

An 82-year-old woman with a height of 152 cm and a weight of 56.6 kg was admitted as an emergency case to our hospital with an open fracture of the distal end of the right femur. Until the day of admission, she was taking 10 mg of empagliflozin (an SGLT2i) and 1000 mg of metformin hydrochloride per day for the management of type 2 diabetes. On admission, the patient's glycemic control was poor. Her condition was initially managed with fasting, and the patient received an isotonic crystalloid solution containing 1% glucose at a rate of 60 mL/hour, which was administered intravenously. Oral intake was resumed at noon on the day following admission. No insulin or antidiabetic agents were administered, as the patient's blood glucose levels remained stable after admission (Table 1).

**Table 1:** Intraoperative and postoperative blood test data

Parameter	On Admission Day	Admission to Surgery	After Induction of Anesthesia	1 Hour	2 Hours (End of Surgery)	Upon Admission to Intensive Care Unit (ICU)	2 Hours After Returning to ICU	5 Hours After Returning to ICU	9 Hours After Returning to ICU	17 Hours After Returning to ICU	Reference Range
HbA1c (%)	7.8	-	-	-	-	-	-	-	-	-	4.6–6.2
(Peripheral blood) Glu (mg/dL)	210	100–160	-	-	-	-	-	-	-	-	70–105
(Arterial blood) pH	-	-	7.396	7.376	7.337	7.356	7.375	7.441	7.423	7.445	7.35–7.45
(Arterial blood) pO₂ (mmHg)	-	-	137	110	98.5	72.4	92.7	72.9	73.6	72.6	83–108
(Arterial blood) pCO₂ (mmHg)	-	-	36	40.1	43.6	42.8	41.3	34.7	40.8	42.6	35–45
(Arterial blood) HCO₃⁻ (mmol/L)	-	-	21.6	23	22.7	23	23.6	24.4	26.2	28.5	22–26
(Arterial blood) Na (mEq/L)	-	-	134	134	133	131	131	132	131	130	135–145
(Arterial blood) K (mEq/L)	-	-	3.9	3.4	3.9	3.8	4.3	4.1	4.4	3.7	3.5–5.0
(Arterial blood) Glu (mg/dL)	-	-	126	118	150	141	125	115	116	110	70–105
(Arterial blood) Lac (mmol/L)	-	-	0.8	0.6	1.1	0.7	1	0.8	0.8	0.7	0.4–1.6
(Peripheral blood) Beta-hydroxybutyrate (mmol/L)	-	-	3.2	2	0.6	-	-	-	-	-	Normal: <0.6 mmol/L; Ketonemia: ≥3.0 mmol/L

**Figure 1:**
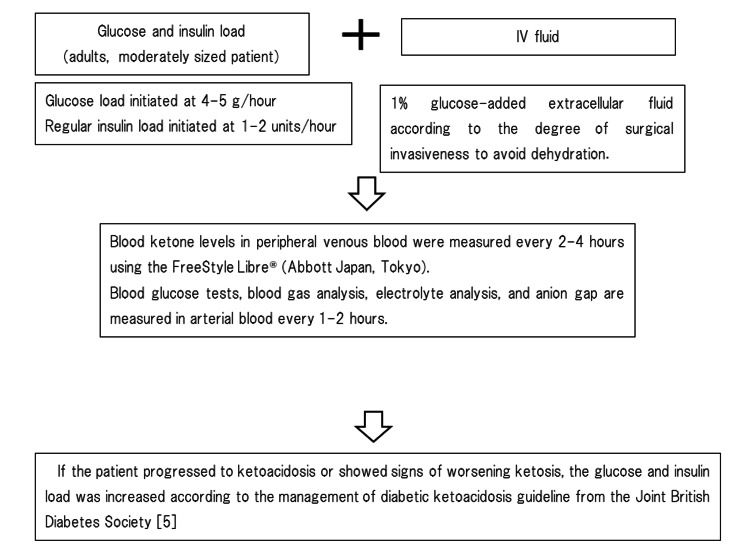
Intraoperative regimen for managing patients with inadequate durations of preoperative SGLT2i discontinuation SGLT2i: sodium-glucose cotransporter 2 inhibitor Image created by the authors.

Two days after admission, the patient underwent open reduction and internal fixation for her bone fracture. Due to an insufficient preoperative withdrawal period of empagliflozin, which increases the risk of perioperative ketoacidosis, we implemented an intraoperative management regimen developed at our institution. This regimen involved monitoring BHB levels using the FreeStyle Libre® system (Abbott Japan, Tokyo, Japan) and adjusting the glucose and insulin administration accordingly (Figure 1). A blood ketone concentration of 3 mmol/L or higher fulfills one of the established diagnostic criteria for diabetic ketoacidosis [5].

Upon admission to the operating room, no clinical signs suggesting ketoacidosis were observed, and vital signs were as follows: blood pressure 154/65 mmHg, heart rate 73 bpm, SpO_2_ 96% (room air), and body temperature 37.5℃. Anesthesia was induced with remimazolam (0.1 mg/kg) and rocuronium (0.7 mg/kg), with remifentanil administered at 0.15 µg/kg/min. Anesthesia was maintained with remimazolam (0.27-0.5 mg/kg/hour), fentanyl (200 µg), remifentanil (0.03-0.18 µg/kg/min), rocuronium, and a right fascia iliaca compartment block. In addition to peripheral blood BHB levels, arterial blood glucose, blood gas, and electrolyte levels were measured hourly during anesthetic management using samples obtained from an indwelling arterial line placed for continuous invasive arterial blood pressure monitoring. After the induction of anesthesia, the peripheral blood BHB concentration was elevated, indicating a state generally considered to be ketonemia [5]. However, arterial blood glucose, pH, and HCO₃⁻ levels were normal, indicating normoglycemic ketonemia.

To prevent progression to EDKA, the patient was managed according to our trial regimen, which included continuous intravenous administration of glucose at a rate of 88 mg/kg/hour, regular insulin at 2 units/hour, and intravenous fluid replacement. To prevent dehydration, a 1% dextrose-containing isotonic crystalloid solution was also administered intravenously. Peripheral blood BHB levels tended to decrease during the operation. Blood glucose, pH, HCO₃⁻, and potassium levels were within normal limits throughout the entire course of the procedure. At the end of the surgery, the insulin and glucose infusions were discontinued as the peripheral blood BHB level had returned to within the normal range. The surgery time was 1 hour 47 minutes, the anesthesia time was 3 hours 7 minutes, and the estimated blood loss was 166 mL. The following intravenous infusions were administered: 600 mL of a glucose-free isotonic crystalloid solution, 1,400 mL of a 1% dextrose-containing isotonic crystalloid solution (14 g of dextrose), 200 mL of a 5% dextrose-containing isotonic crystalloid solution (25 g of dextrose), and 4 units of packed red blood cells. The total dose of regular insulin was 4.2 units. On the day of surgery, the patient fasted and received 1,230-1,440 mL/day of 5% glucose-containing isotonic crystalloid solution as both supplementation and maintenance therapy, providing 21.3-38.1 g/day of glucose. Regular insulin was continuously administered at a rate of 0.5 units/hour.

On postoperative day 1, oral intake was resumed, and the patient was able to consume full meals; therefore, both intravenous fluid infusion and continuous insulin administration were discontinued. Arterial blood gas analysis on postoperative days 0 and 1 revealed that blood glucose, pH, and HCO₃⁻ levels remained within the normal limits. Beginning on postoperative day 3, insulin was administered according to a sliding scale regimen.

On postoperative day 7, the dipeptidyl peptidase-4 inhibitor linagliptin (5 mg/day) was started orally as a treatment for diabetes mellitus. Empagliflozin and metformin were not resumed during hospitalization, and no symptoms of ketoacidosis were observed. The patient was transferred to a rehabilitation facility on postoperative day 19.

## Discussion

Patients who receive SGLT2i are at risk for hyperketonemia if surgical stress increases insulin resistance or decreases secretion [6]. The half-life of SGLT2is is approximately 5-18 hours [7], and the U.S. Food and Drug Administration recommends SGLT2i withdrawal 3-4 days prior to surgery to prevent the onset of DKA [8]. A total of 2.03 per 1000 patients with type 2 diabetes are reported to develop DKA annually [9], and approximately 35-71% of DKA cases are EDKA [10]. EDKA can occur in the perioperative period and can lead to coma in severe cases [11]. Other perioperative risk factors for EDKA include fasting and fluid restriction, as well as the associated reduction or discontinuation of insulin [4]. EDKA can be challenging to diagnose in clinical settings, as it is not associated with significant hyperglycemia (blood glucose >250 mg/dL), which is typically relied upon for the diagnosis of diabetic ketoacidosis [12]; additionally, delayed diagnosis has been reported in 50% of SGLT2i-associated EDKA cases [13]. Although the typical symptoms of DKA, including dyspnea, nausea, and vomiting [14], may aid in the diagnosis of conscious patients, these symptoms cannot be assessed under general anesthesia. In the present case, asymptomatic, normoglycemic ketonemia was identified preoperatively by measuring the peripheral blood BHB levels. Intraoperative management was conducted using a previously prototyped regimen specifically developed for patients receiving SGLT2is.

Glucose and insulin administration is recommended to prevent EDKA in patients with insufficient preoperative SGLT2i discontinuation [4]. However, due to interindividual variability in insulin resistance, the glycemic and ketone responses to this approach are often unpredictable, even when the drug has been withdrawn.

The optimal regimen for intraoperative management remains unknown. We developed an insulin and glucose regimen for intraoperative management based on prior literature. The amount of glucose excreted in urine from patients who are treated with SGLT2is is reported to be approximately 60-80 g per day [15]. Specifically, the oral administration of empagliflozin has been shown to result in urinary glucose excretion of up to 100-120 g per day [16]. Therefore, the initial intraoperative glucose load starting dose was 5 g/hour. In addition, an extracellular fluid supplement containing 1% dextrose, which has been reported to prevent both hyperglycemia and hypoglycemia while suppressing intraoperative ketone body production [17], was administered according to the surgical invasiveness.

The protocol for DKA that was proposed by Kitabchi et al. and endorsed by the American Diabetes Association [11] was developed for the management of DKA with significant hyperglycemia. The insulin dosage recommended in the protocol (0.02-0.05 units/kg/hour) is typically administered during DKA treatment when blood glucose levels have stabilized and corresponds approximately to the physiological basal insulin secretion rate (0.5-1.0 units/hour), which is considered a relatively safe starting point for patients with unknown insulin requirements [18]. To further ensure the safety of the procedure, we assessed whether the glucose and insulin doses were appropriate based on peripheral blood BHB levels. Depending on intraoperative peripheral blood BHB levels and the trend in blood glucose levels, the loading rates of insulin and glucose were modified. If progression to ketonemia or ketoacidosis was observed, the guidelines for treating EDKA according to the Joint British Diabetes Society [5] were followed. In this patient, BHB measurement in peripheral blood was useful for assessing intraoperative insulin and glucose loading requirements. The intraoperative management strategy used for this patient may help accommodate interindividual variability in insulin resistance; however, further investigation is needed to validate its safety and effectiveness.

There are three types of ketone bodies: acetoacetic acid, BHB, and acetone [19]. There are two types of ketone measurements: urine and blood tests. The urine test, although simple, is not suitable for intraoperative DKA screening because it primarily measures acetoacetic acid [5], which may be underestimated, as BHB, which is the major ketoacid in DKA, is not detected. It is recommended that blood ketone levels rather than urine ketone levels be measured when a patient receiving SGLT2i requires emergency surgery [20]. At our hospital, a device for measuring blood ketone levels, originally used to manage homebound patients with type 1 diabetes, was available and incorporated into our intraoperative management regimen. The peripheral BHB analyzer used for this patient allowed for simple, rapid, and sensitive ketone detection. In this patient, peripheral blood BHB measurement proved useful for intraoperative management while the patient received empagliflozin therapy. Although it would have been ideal to continue BHB monitoring postoperatively through to the following day, the BHB level had normalized by the end of surgery. Therefore, postoperative management was continued with reference to the intraoperative glucose and insulin doses, and patient monitoring was based on clinical symptoms and arterial blood gas analysis.

## Conclusions

Emergency surgery in patients receiving SGLT2is with insufficient drug discontinuation durations poses the risk of perioperative DKA, yet specific intraoperative management strategies remain poorly defined. In this case, the use of a prototype regimen of insulin and glucose administration, with reference to regular BHB measurements, successfully prevented progression from normoglycemic ketosis to ketoacidosis. This approach may provide a practical option for intraoperative management in similar high-risk settings, warranting further validation in future studies.

## References

[1] Gallo LA, Wright EM, Vallon V (2015). Probing SGLT2 as a therapeutic target for diabetes: basic physiology and consequences. Diab Vasc Dis Res.

[2] Qiu H, Novikov A, Vallon V (2017). Ketosis and diabetic ketoacidosis in response to SGLT2 inhibitors: basic mechanisms and therapeutic perspectives. Diabetes Metab Res Rev.

[3] Fadini GP, Bonora BM, Avogaro A (2017). SGLT2 inhibitors and diabetic ketoacidosis: data from the FDA Adverse Event Reporting System. Diabetologia.

[4] Meyer EJ, Gabb G, Jesudason D (2018). SGLT2 inhibitor-associated euglycemic diabetic ketoacidosis: a South Australian clinical case series and Australian spontaneous adverse event notifications. Diabetes Care.

[5] Dhatariya KK (2022). The management of diabetic ketoacidosis in adults—an updated guideline from the Joint British Diabetes Society for Inpatient Care. Diabet Med.

[6] Rosenstock J, Ferrannini E (2015). Euglycemic diabetic ketoacidosis: a predictable, detectable, and preventable safety concern with SGLT2 inhibitors. Diabetes Care.

[7] Saisho Y (2020). SGLT2 inhibitors: the star in the treatment of type 2 diabetes?. Diseases.

[8] (2024). FDA revises labels of SGLT2 inhibitors for diabetes to include warnings about too much acid in the blood and serious urinary tract infections. https://www.fda.gov/drugs/drug-safety-and-availability/fda-revises-labels-sglt2-inhibitors-diabetes-include-warnings-about-too-much-acid-blood-and-serious.

[9] Douros A, Lix LM, Fralick M (2020). Sodium-glucose cotransporter-2 inhibitors and the risk for diabetic ketoacidosis: a multicenter cohort study. Ann Intern Med.

[10] Bonora BM, Avogaro A, Fadini GP (2018). Sodium-glucose co-transporter-2 inhibitors and diabetic ketoacidosis: an updated review of the literature. Diabetes Obes Metab.

[11] Kitabchi AE, Umpierrez GE, Miles JM, Fisher JN (2009). Hyperglycemic crises in adult patients with diabetes. Diabetes Care.

[12] Modi A, Agrawal A, Morgan F (2017). Euglycemic diabetic ketoacidosis: a review. Curr Diabetes Rev.

[13] Dizon S, Keely EJ, Malcolm J, Arnaout A (2017). Insights into the recognition and management of SGLT2-inhibitor-associated ketoacidosis: it's not just euglycemic diabetic ketoacidosis. Can J Diabetes.

[14] Seki H, Ideno S, Shiga T (2023). Sodium-glucose cotransporter 2 inhibitor-associated perioperative ketoacidosis: a systematic review of case reports. J Anesth.

[15] Ahmadieh H, Ghazal N, Azar ST (2017). Role of sodium glucose cotransporter-2 inhibitors in type I diabetes mellitus. Diabetes Metab Syndr Obes.

[16] Al Jobori H, Daniele G, Adams J (2018). Empagliflozin treatment is associated with improved β-cell function in type 2 diabetes mellitus. J Clin Endocrinol Metab.

[17] Yamasaki K, Inagaki Y, Mochida S, Funaki K, Takahashi S, Sakamoto S (2010). Effect of intraoperative acetated Ringer's solution with 1% glucose on glucose and protein metabolism. J Anesth.

[18] Bolli GB, Home PD, Porcellati F (2025). The modern role of basal insulin in advancing therapy in people with type 2 diabetes. Diabetes Care.

[19] Michaels GD, Margen S, Liebert G, Kinsell LW (1951). Studies in fat metabolism. I. The colorimetric determination of ketone bodies in biological fluids. J Clin Invest.

[20] (2024). Perioperative care of people with diabetes undergoing surgery. https://cpoc.org.uk/guidelines-and-resources/guidelines/guideline-diabetes.

